# Causal Link of Seven Trace Elements and Eight Nutrients With Meningioma: A Bidirectional Two‐Sample Mendelian Randomization Analysis

**DOI:** 10.1002/brb3.70852

**Published:** 2025-09-02

**Authors:** Yihong Hao, Jieqiong Ren, Jie Han, Dajiang Hao

**Affiliations:** ^1^ Department of Neurosurgery The Third People's Hospital of Datong Datong China

**Keywords:** Mendelian randomization, meningioma, nutrients, trace elements, zinc

## Abstract

**Background:**

Meningiomas, the second most common intracranial tumors, account for over one‐third of primary central nervous system tumors. Recent studies suggest a link between trace elements, nutrients, and tumor development. This study used Mendelian randomization (MR) to investigate causal links between trace elements, nutrients, and meningioma.

**Methods:**

The data for trace elements and nutrients were derived from the published IEU OpenGWAS database. The genome‐wide association study data for meningiomas were sourced from the Finnish database. Two‐sample MR approach was leveraged to examine the causal link of seven trace elements and eight nutrients with meningioma. Inverse‐variance weighted (IVW), MR‐Egger test, and weighted median methods were applied to infer causal links. Sensitivity analyses were performed to examine the robustness of the results. Reverse MR (RMR) analysis was further carried out with meningiomas as the exposure factor and trace elements and nutrients as the outcome. A multivariable MR (MVMR) method was adopted to appraise the combined effects of several exposure factors on meningioma.

**Results:**

The two‐sample MR analysis revealed that Zn (OR = 1.484, 95% CI: 1.222–1.803, *p* = 6.90E‐05), as an exposure factor, may increase the risk of meningioma. The RMR analysis indicated no evident causal connection. According to the MVMR analysis, Zn was an independent risk factor for meningiomas (OR = 1.391, 95% CI: 0.042–0.617, *p* = 0.025).

**Conclusion:**

Zn is an independent risk factor for meningioma, which is significant for developing prevention and management strategies for meningioma.

## Graphical abstract

This Mendelian randomization study explored the causal associations of seven trace elements and eight nutrients with the risk of meningioma using the IEU OpenGWAS and FinnGen databases. Two‐sample MR and multivariate MR analyses revealed zinc (Zn) as an independent risk factor for meningioma (IVW OR = 1.484, 95% CI 1.222–1.803, *p* = 6.90 × 10^−5^; MVMR OR = 1.391, 95% CI 1.042–1.617, *p* = 0.025). These findings provide genetic evidence, establishing an association between zinc exposure and meningioma and inform preventive strategies.

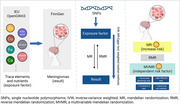

## 1 Background

Apart from gliomas, meningioma is the most frequently occurring tumor in the central nervous system (Christine et al. 2008). Statistical data indicate that it accounts for roughly 36.8% of all central nervous system tumors (Ostrom et al. 2017). The incidence of meningiomas rises with age, with adults over 65 being the most affected group (Goldbrunner et al. 2021). The female‐to‐male ratio is approximately 2:1 (Nazem et al. 2020). Meningiomas are tumors that develop from the cells of the meninges (arachnoid membrane), and the majority of them are benign (Ogasawara et al. 2021). They usually grow slowly and may not cause any symptoms for many years, or the symptoms may be mild, making them easy to overlook. The clinical manifestations of meningioma patients depend on the location and size of the tumor. The typical clinical symptoms include elevated intracranial pressure, focal neurological deficits (including cranial nerve involvement), and generalized or partial seizures caused by focal mass effects. The most common symptoms are headache, seizures, visual disturbances, limb weakness, and changes in mental status.

Previous studies have identified risk factors for meningioma development, including sex, smoking, and radiation exposure (Flint‐Richter et al. 2011). Recent research has further suggested potential roles for breast cancer (Huang et al. 2025) and inflammatory factors (Z. Zhang et al. 2023) in meningioma pathogenesis. Even though surgery resection is the current standard treatment, approximately 20% of meningiomas are aggressive and prone to recurrence (Domingues et al. 2014; Ostrom et al. 2022). Hence, identifying relevant influencing factors can support clinicians in delivering timely, effective, and personalized treatment to meningioma patients.

Despite being present in small quantities, trace elements and nutrients are essential for various physiological functions in the human body. Most trace elements and nutrients serve as coenzymes for biological enzymes within the body. They are crucial for maintaining enzyme activity, regulating acid‐base balance and osmotic pressure of bodily fluids, as well as sustaining physiological functions and biochemical activities of cells (H. Yang et al. 2007). Recent research has revealed that during the onset and development of diseases, abnormal changes in the levels of trace elements and nutrients are noticed, indicating that the measurement of these levels may help in disease diagnosis, treatment, and monitoring (Cao et al. 2024). A previous study has indicated that changes in the levels of trace elements and nutrients can directly or indirectly affect the natural progression of certain cancers by influencing various biological processes like cellular metabolism (Jayaraman and Jayaraman 2011). Moreover, certain studies have indicated that trace elements and nutrients might be linked to the prevention or incidence of specific brain tumors (Hrabeta et al. 2016; W. Zhang et al. 2022). Nevertheless, there remains a scarcity of high‐quality evidence to elucidate the causal relationship between certain trace elements or nutrients and the pathogenesis of meningiomas.

Strictly designed randomized controlled trials are considered the gold standard for determining causal links and effectively identifying potential confounders. Nevertheless, the process of conducting randomized controlled trials is extremely difficult due to ethical restrictions, challenges in double‐blind design, interference from both internal and external factors, limited statistical power, and significant cost and time requirements. With the increasing number of genome‐wide association studies (GWAS), Mendelian randomization (MR) has proven to be an effective tool for causal inference across various phenotypes (Zhu et al. 2023). MR is an epidemiological study design and data analysis method that employs Mendel's law of independent assortment to test causal assumptions (Davies et al. 2018). Through the random distribution of alleles during gamete formation, MR estimates the effect of genotype on disease outcomes. This approach is less likely to encounter confounders and reverse causality, which are frequently observed in traditional epidemiological research (Bowden and Holmes 2019). Additionally, multivariable MR (MVMR) extends MR by considering the causal associations between several exposure factors and diseases. This method allows MVMR studies to appraise the joint impact of multiple exposure factors on disease risk, as well as the interactions between these exposure factors (Sanderson 2021; Sanderson et al. 2020). Hence, the purpose of this study is to employ MR to examine the causal links of seven trace elements and eight nutrients with meningiomas, in order to provide scientifically valid treatment recommendations for meningioma patients.

## 2 Materials and Methods

### 2.1 Study Design

**FIGURE 1:**
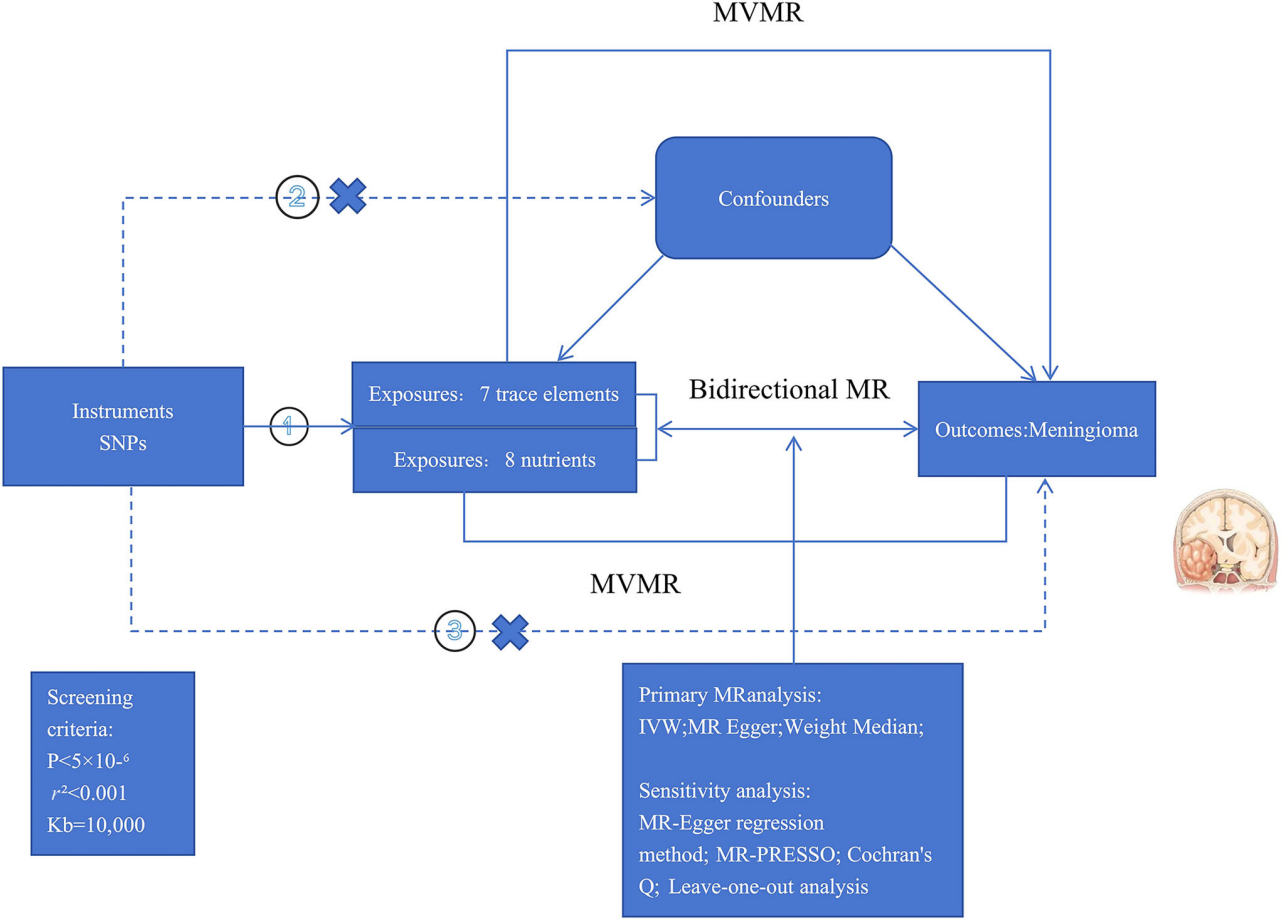
Flow chart of the Mendelian randomization study of trace elements and meningiomas.

This study leveraged seven trace elements (copper [Cu], calcium [Ca], iron, zinc [Zn], magnesium, potassium, selenium [Se]) and eight nutrients (folic acid, carotene, vitamin A, vitamin B12, vitamin B6 [VB6], vitamin C, vitamin D, and vitamin E) as exposure factors, with meningiomas as the outcome, to carry a two‐sample MR analysis. Additionally, meningioma was utilized as the exposure factor, with trace elements and nutrients as outcomes, to perform a reverse MR (RMR) analysis. Finally, seven trace elements and eight nutrients were integrated as composite exposure factors for MVMR analysis to evaluate the combined impact of multiple exposures on meningiomas. Figure 1 presents the three main assumptions of MR. Assumption 1 stated that the selected single nucleotide polymorphisms (SNPs) were notably linked to the exposure factors. Assumption 2 was that these SNPs must be independent of any potential confounders between the exposure and the outcome. Assumption 3 proposed that although these SNPs were not directly tied to meningiomas, a causal link through the exposure factors could be established. This study adhered to the strengthening the reporting of observational studies in epidemiology using MR: the STROBE‐MR statement (Skrivankova et al. 2021).

### 2.2 GWAS Data Collection for Exposure Factors and Outcome Events

The present study utilized publicly accessible GWAS datasets.

#### 2.2.1 Sources of GWAS Data for Trace Elements and Nutrients

Exposure data for Cu, Se, and Zn were obtained from a cross‐population GWAS conducted by Evans et al. (2013). The research integrated genome‐wide association analyses from Australian adults and UK pregnant women cohorts, employing inductively coupled plasma mass spectrometry for measurement. It identified genes significantly linked to blood levels of Cu, Se, and Zn. Exposure data for the remaining 12 trace elements, including Ca, β‐carotene, and folate, were derived from a GWAS of 500,000 European individuals conducted by the UK Biobank (Bycroft et al. 2018). Serum/plasma marker concentrations were measured using standardized platforms (colorimetry, mass spectrometry, etc.). The data, following rigorous quality control, were released on the IEU OpenGWAS database. These data are accessible via the IEU OpenGWAS database (https://gwas.mrcieu.ac.uk, with IDs listed in Table 1).

**TABLE 1:** Data sources and demographics.

Exposures or outcome	Sample size	Ancestry	Sex	Significance level	GWAS ID or URL	No. of SNPs
Calcium	64,979	European	Males and females	5e‐6	ukb‐b‐8951	9,851,867
Carotene	64,979	European	Males and females	5e‐6	ukb‐b‐16202	9,851,867
Copper	2603	European	Males and females	5e‐6	ieu‐a‐1073	2,543,646
Folate	64,979	European	Males and females	5e‐6	ukb‐b‐11349	9,851,867
Iron	64,979	European	Males and females	5e‐6	ukb‐b‐20447	9,851,867
Magnesium	64,979	European	Males and females	5e‐6	ukb‐b‐7372	9,851,867
Potassium	64,979	European	Males and females	5e‐6	ukb‐b‐17881	9,851,867
Selenium	2603	European	Males and females	5e‐6	ieu‐a‐1077	2,543,646
Vitamin A	460,351	European	Males and females	5e‐6	ukb‐b‐9596	9,851,867
Vitamin B12	64,979	European	Males and females	5e‐6	ukb‐b‐19524	9,851,867
Vitamin B6	64,979	European	Males and females	5e‐6	ukb‐b‐7864	9,851,867
Vitamin C	64,979	European	Males and females	5e‐6	ukb‐b‐19390	9,851,867
Vitamin D	64,979	European	Males and females	5e‐6	ukb‐b‐18593	9,851,867
Vitamin E	64,979	European	Males and females	5e‐6	ukb‐b‐6888	9,851,867
Zinc	2603	European	Males and females	5e‐6	ieu‐a‐1079	2,543,610
Meningioma	345,737	European	Males and females	5e‐8	www.finngen.fi/en	1,048,575

#### 2.2.2 Meningioma GWAS Data Source

The Finnish Genetic Study (FinnGen) combines GWAS data collected from Finnish biobanks with digital health records from the Finnish health registry system. Summary statistics for meningioma were extracted from FinnGen's R9 database release. This dataset encompassed 345,737 individuals of European ancestry, of whom 1605 were cases and 344,132 were controls. Meningioma classification was based on the International Classification of Diseases, Tenth Revision (ICD‐10) codes from participants' hospital records. These data, ethically reviewed and publicly available, can be accessed on the FinnGen platform (FinnGen: An Expedition Into Genomics and Medicine n.d.; Kurki et al. 2023).

### 2.3 Screening of Instrumental Variables (IVs)

In this study, the threshold for the significance of the correlation *p* value for IVs was set at *p* < 5 × 10^−6^. This criterion was established based on high‐quality MR studies and was applied to select IVs related to trace elements and nutrients (X. Liu et al. 2023; Su et al. 2023; Xia et al. 2023). Statistically significant SNP loci from the GWAS summary data of trace elements and nutrients were chosen as the initial screened IVs. Additionally, a linkage disequilibrium coefficient *r* ^2^ was set at 0.001, with a region width of 10,000 KB, to minimize the impact of pleiotropy on the results (Kurilshikov et al. 2021). The F‐statistic was computed to appraise whether the selected IVs was weak, utilizing the formula *F* = (*N* − *K* − 1/*K*) × (*R*2/1 − *R*2). *R*2 represented the extent to which the IVs explained the exposure, serving as the coefficient of determination in the regression equation. *N* was the number of samples in the exposure GWAS study. *K* was the number of IVs (Palmer et al. 2012). The calculation of *R*2 was done by the following formula: *R*2 = 2 × β2 × EAF × (1 − EAF)/[2 × β2 × EAF × (1 − EAF) + SE2 × 2× N × EAF(1 − EAF)]. If the F‐statistic exceeded 10, it indicated that there was no potential bias of weak IVs. IVs with an F‐statistic less than 10 should be excluded (Li et al. 2022).

### 2.4 Two‐Sample MR Analysis

Inverse‐variance weighted (IVW) was leveraged as the primary method for MR analysis, with MR‐Egger and weighted median used as supplementary methods. The IVW method operates by utilizing the inverse of the variance of each IVs as its weight, assuming that all IVs are valid. The regression excluded the intercept term, and the final result was the weighted mean of all effect sizes. When the IVs satisfied the three basic assumptions, it achieved higher estimation accuracy and test power. In the absence of heterogeneity and pleiotropy, it was preferable to utilize the IVW estimates, as this method provided estimates with a higher degree of reliability (Choudhury et al. 2022; Pierce and Burgess 2013).

### 2.5 Sensitivity Analysis

Several sensitivity and validation analyses were carried out to appraise the potential biases in the MR analysis. Horizontal pleiotropy between the IVs was appraised by means of the MR‐Egger regression method. If the MR‐Egger intercept equaled 0 or p > 0.05, it was suggested that horizontal pleiotropy may not be present (Bowden et al. 2017). If pleiotropy was observed, the weighted median method should be used as the preferred approach (Burgess et al. 2017). Outliers were detected utilizing the MR pleiotropy residual sum and outlier (MR‐PRESSO) method. If outliers presented, they were removed, and the analysis was conducted again. Cochran's *Q* test was leveraged to appraise the heterogeneity of the IVs. If the *p*‐value exceeded 0.05, it was assumed there was no heterogeneity. Leave‐one‐out (LOO) analysis is a method that tests whether the removal of a single SNP affects the link between exposure and outcome. By excluding each SNP one by one, it helps assess the robustness of the findings.

### 2.6 MVMR Analysis

The MVMR method is applicable to multiple genetic instruments, regardless of their connection to the exposure factors (Sanderson et al. 2019). In this approach, although IVs may be linked to multiple risk factors, they must meet the equivalent IVs assumption (Burgess et al. 2016). Thus, the MVMR method was leveraged to analyze the causal links between various exposure factors and diseases, to evaluate the joint impact of multiple exposures on disease risk and their interactions.

### 2.7 General Criteria for Evaluating the Significance of Results

To mitigate the problem of multiple testing in two‐sample MR analysis, we applied the Bonferroni correction significance threshold, which resulted in a value of 3.3 × 10^−3^ (for 15 tests, 0.05/15). If the estimates from the three methods (IVW, MR‐Egger, and weighted median) were consistent in direction, the IVW method passed the significance threshold, and neither the MR‐Egger intercept test nor the MR‐PRESSO global test detected notable pleiotropy, the result was considered significant. In MVMR analysis, the p value threshold was leveraged to appraise the significance of the link between exposure factors and outcomes. If *p* < 0.05, it indicated that the link between the exposure factors and outcomes was statistically significant. The TwoSampleMR (0.6.17) and MendelianRandomization (0.10.0) packages in R version 4.4.2 were leveraged for all analyses.

## 3 Results

### 3.1 Selection of IVs

The selection of SNPs linked to trace elements and nutrients was conducted utilizing a significance threshold of *p* < 5 × 10^−6^. After removing confounders and SNPs tied to trace elements and nutrients, the final set of SNPs was determined. The number of SNPs tied to exposure to various trace elements and nutrients ranges from 6 to 19 (Table 2). Based on the F‐statistic, weak IVs were removed. The F‐statistic range was calculated to be 20.867–84.683, with all *F*‐values >10, indicating that the selected IVs exhibited strong genetic variation (Supporting Information).

**TABLE 2:** Sensitivity analysis of trace elements and nutrients on meningiomas utilizing a two‐sample MR approach.

Outcome	Exposure	SNP	Heterogeneity tests		Directional horizontal pleiotropy test	
			Methods	Cochran's Q (*p*)	MR‐Egger intercept (*p*)	Pleiotropy^a^ (*p*)
Meningioma	Calcium	19	MR‐Egger, IVW	29.257 (0.032), 31.805 (0.023)	0.240	0.071
Meningioma	Carotene	16	MR‐Egger, IVW	7.129 (0.930), 8.027 (0.921)	0.359	0.917
Meningioma	Copper	6	MR‐Egger, IVW	7.959 (0.093), 8.136 (0.149)	0.780	0.339
Meningioma	Folate	14	MR‐Egger, IVW	7.368 (0.832), 7.372 (0.882)	0.947	0.880
Meningioma	Iron	13	MR‐Egger, IVW	5.840 (0.828), 5.844 (0.884)	0.951	0.889
Meningioma	Magnesium	18	MR‐Egger, IVW	26.480 (0.048), 28.623 (0.038)	0.272	0.057
Meningioma	Potassium	15	MR‐Egger, IVW	30.186 (0.003), 30.236 (0.004)	0.891	0.816
Meningioma	Selenium	6	MR‐Egger, IVW	2.063 (0.559), 2.064 (0.724)	0.984	0.804
Meningioma	Vitamin A	11	MR‐Egger, IVW	11.644 (0.234), 15.859 (0.104)	0.105	0.107
Meningioma	Vitamin B6	17	MR‐Egger, IVW	14.126 (0.440), 14.162 (0.513)	0.853	0.562
Meningioma	Vitamin B12	9	MR‐Egger, IVW	3.109 (0.875), 4.598 (0.800)	0.262	0.735
Meningioma	Vitamin C	10	MR‐Egger, IVW	18.106 (0.020), 18.211 (0.033)	0.835	0.058
Meningioma	Vitamin D	12	MR‐Egger, IVW	11.818 (0.224), 12.260 (0.268)	0.576	0.268
Meningioma	Vitamin E	11	MR‐Egger, IVW	8.317 (0.503), 8.695 (0.561)	0.554	0.628
Meningioma	Zinc	8	MR‐Egger, IVW	2.293 (0.807), 5.054 (0.537)	0.157	0.564

Abbreviations: IVW, inverse‐variance weighted; MR‐Egger, Mendelian randomization‐Egger; MR‐PRESSO, MR pleiotropy residual sum and outlier.

^a^ Detected by MR‐PRESSO global test.

### 3.2 Bidirectional Two‐Sample MR Results

**FIGURE 2:**
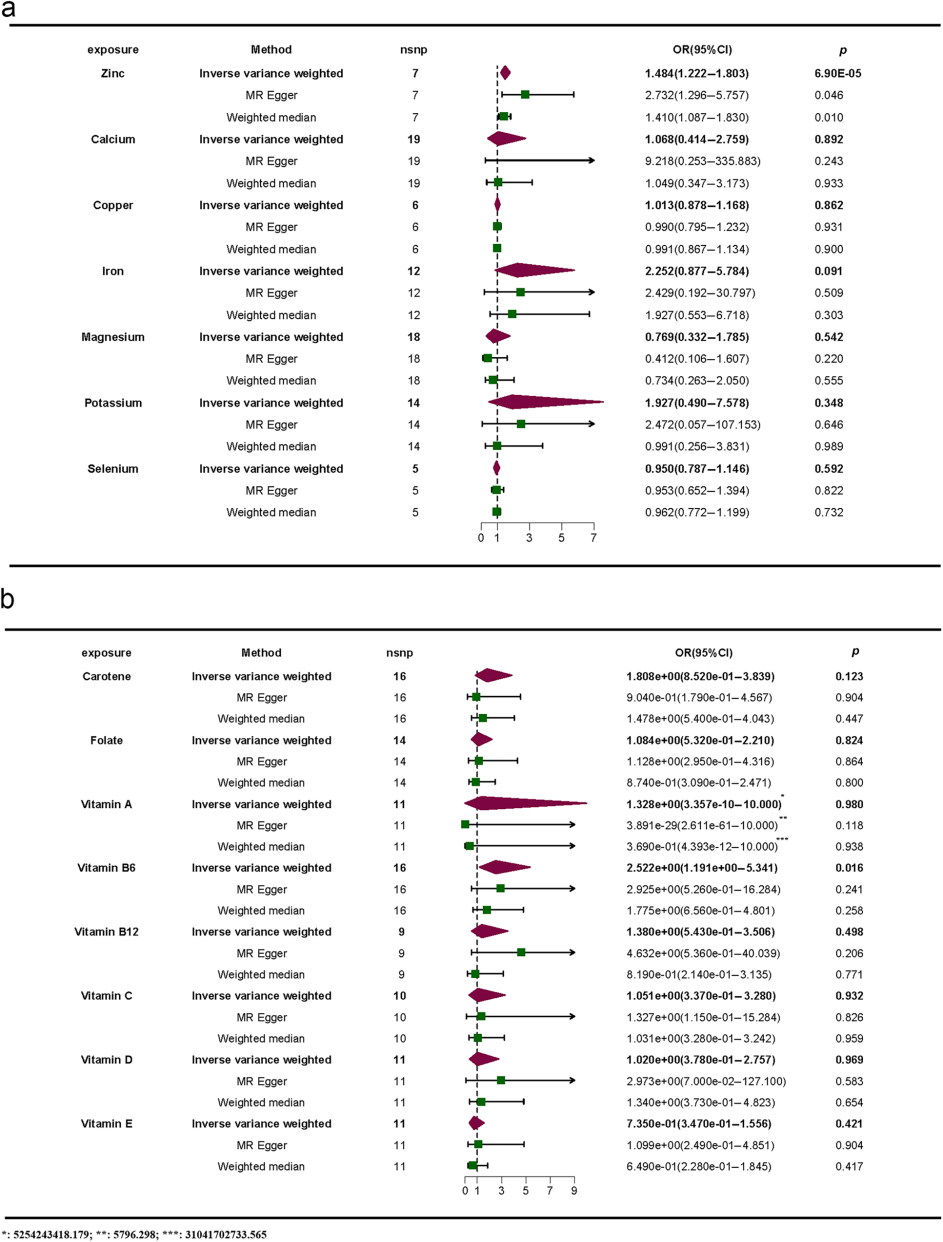
Results of the two‐sample MR analysis: (a) (up) and (b) (down).

**FIGURE 3:**
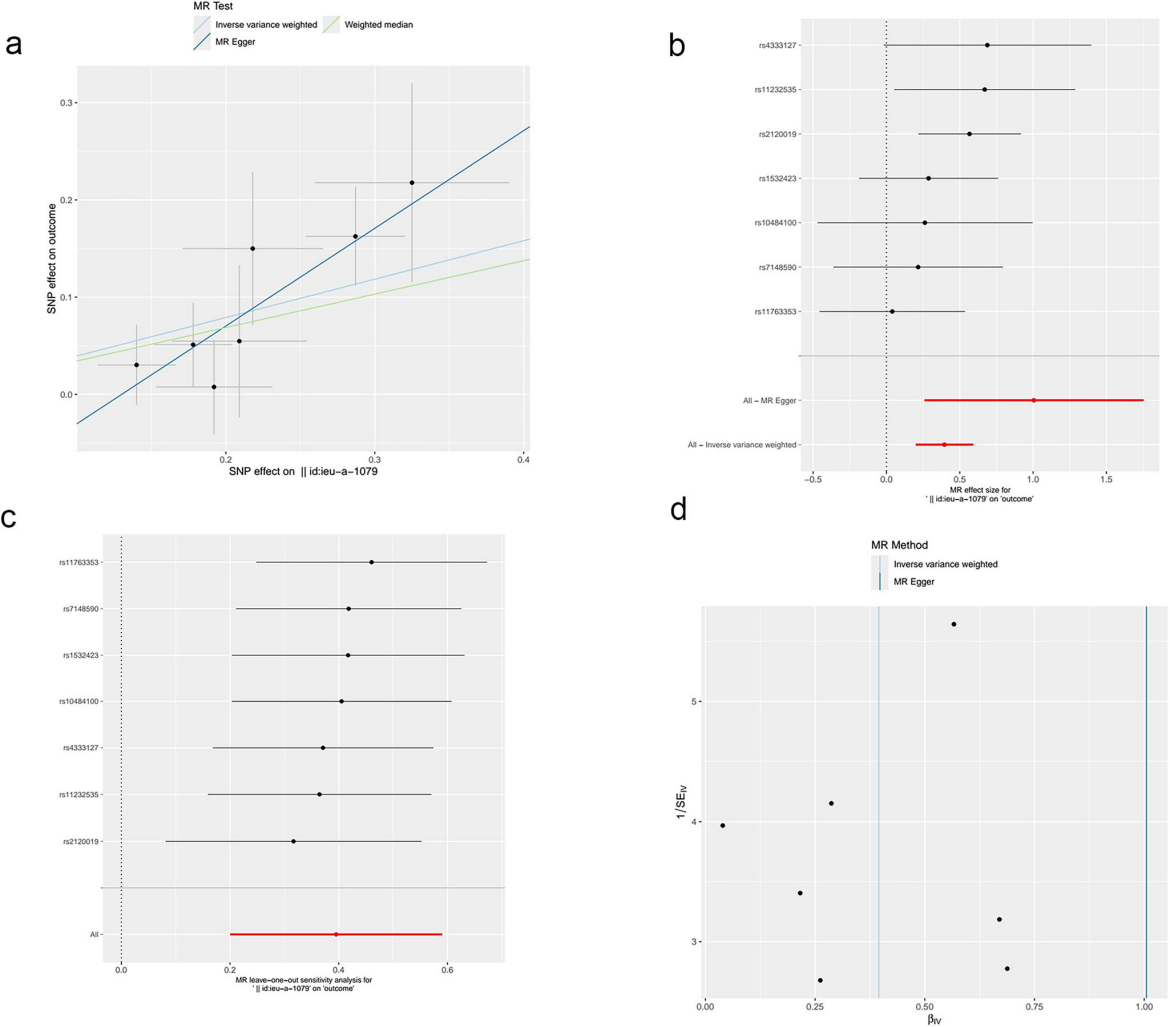
Scatter plot (a), forest plot (b), leave‐one‐out sensitivity analysis (c), and funnel plot (d) of the association of zinc with Meningiomas.

**FIGURE 4:**
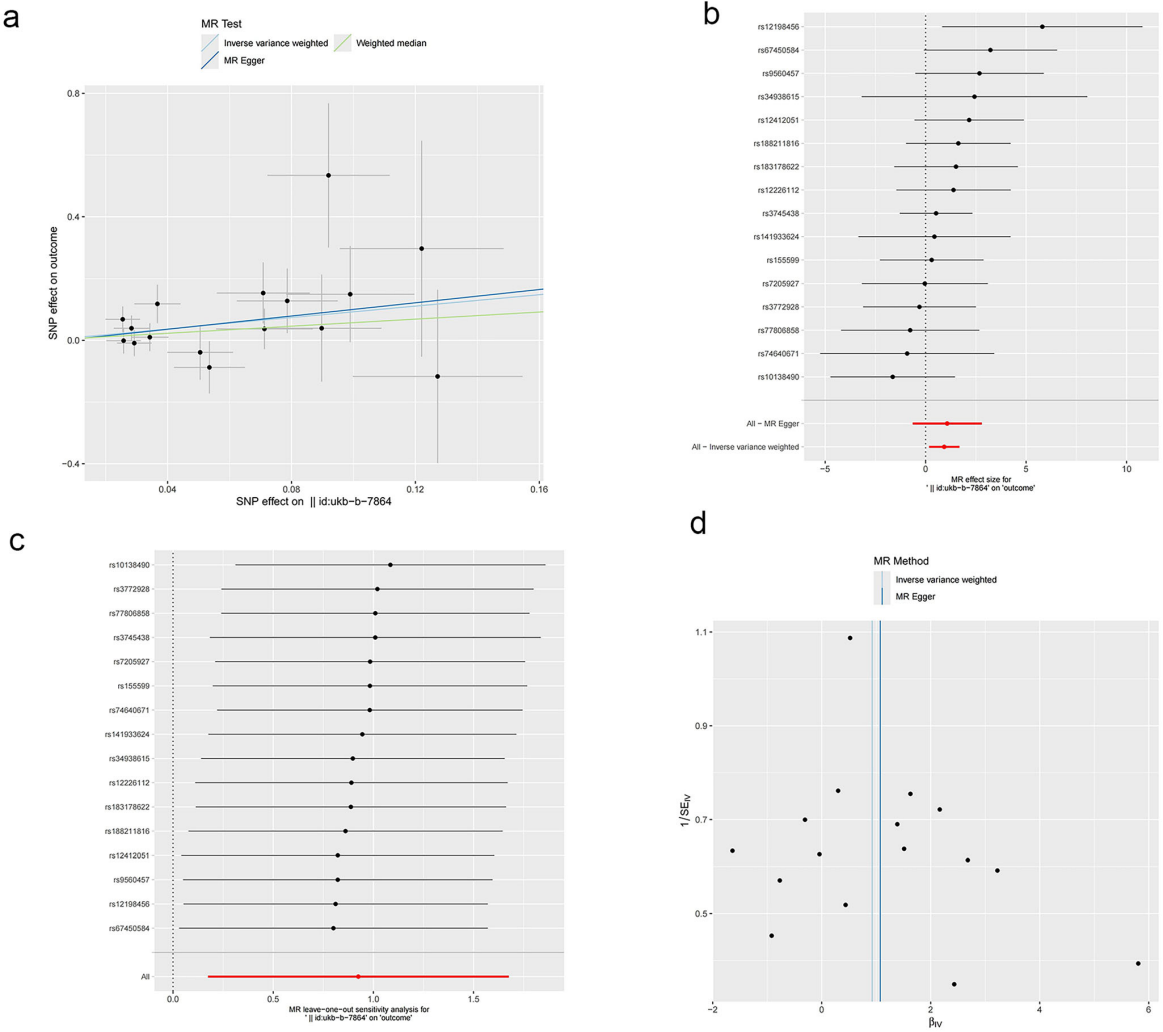
Scatter plot (a), forest plot (b), leave‐one‐out sensitivity analysis (c), and funnel plot (d) of the association of vitamin B6 with Meningioma.

The study employed trace elements and nutrients as exposures, with meningiomas serving as the outcome, to conduct an MR analysis; the results are shown in Figure 2a,b. Before applying Bonferroni correction, the two‐sample MR analysis indicated a positive causal link between Zn (odds ration [OR] = 1.484, 95% confidence interval [CI]: 1.222–1.803, *p* = 6.90E‐05), VB6 (OR = 2.522, 95% CI: 1.191–5.341, *p* = 0.016), and meningioma. Moreover, the beta values from the IVW method, MR‐Egger method, and weighted median method showed consistent directionality (Figures 3a and 4a). However, following Bonferroni correction, the results did not support a positive causal relationship between VB6 and meningiomas. None of the other trace elements or nutrients demonstrated a noticeable causal relationship with meningiomas. No remarkable effect of meningiomas on trace elements or nutrients was noted in RMR analysis, where meningiomas were the exposure and trace elements and nutrients were the outcomes (Supporting Information).

### 3.3 Sensitivity Analysis

To ensure the robustness of the analysis results, MR‐Egger intercept (Zn: *p* = 0.157; VB6: *p* = 0.984), MR‐PRESSO (Zn: *p* = 0.564; VB6: *p* = 0.562), and Cochran's Q (Zn: *Q* = 5.054, *p* = 0.537; VB6: *Q* = 14.162, *p* = 0.513) were leveraged to appraise the pleiotropy and heterogeneity of trace elements and nutrients. No pleiotropy or heterogeneity was detected statistically (p > 0.05). According to the LOO analysis, excluding any individual SNP did not notably impact the causal link estimates, indicating that the MR analysis results were robust (Figures 3c and 4c). The contribution of individual SNPs and the overall estimation effects of Zn and VB6 on meningioma are presented in Figures 3b and 4b. The funnel plot for the MR analysis demonstrated the distribution and intensity of each genetic variation in the link of Zn and VB6 with meningioma (Figures 3d and 4d)

### 3.4 Results of MVMR Analysis

Seven trace elements were employed as exposure factors to appraise their combined influence on disease risk and the interactions among them. The results revealed that Zn still had a direct impact on meningioma (OR = 1.391, 95% CI: 0.042–0.617, *p* = 0.025). The sensitivity analysis demonstrated that no horizontal pleiotropy or heterogeneity was present in any causal effects. The MR analysis results were robust, indicating that Zn could be considered an independent risk factor for meningioma (Table 3). Additionally, under the combined effects of the eight nutrients as exposure factors, no single nutrient was observed to act as an independent risk factor influencing meningiomas (Table 4).

**TABLE 3:** Results of MVMR and sensitivity analysis for trace elements and meningioma.

Exposure	Outcome	Beta	Se	p	OR	95% CI	*Q*	Egger intercept	Pleiotropy test	Heterogeneity test
Calcium	Meningioma	0.151	1.275	0.906	1.163	−2.347 to 2.649	54.131	0.017	0.309	0.001
Copper		−0.031	0.116	0.785	0.969	−0.258 to 0.195				
Iron		2.330	3.158	0.461	10.275	−3.860 to 8.519				
Magnesium		−1.090	5.545	0.844	0.336	−11.959 to 9.779				
Potassium		−1.292	4.460	0.772	0.275	−10.034 to 7.450				
Selenium		−0.219	0.136	0.106	0.803	−0.486 to 0.047				
Zinc		0.330	0.147	0.025	1.391	0.042 to 0.617				

**TABLE 4:** Results of MVMR and sensitivity analysis for nutrients and meningioma.

Exposure	Outcome	Beta	Se	OR	95% CI	*Q*	Egger intercept	Pleiotropy test	Heterogeneity test
Carotene	Meningioma	0.435	0.943	1.545	0.243–9.809	46.147	−0.021	0.201	0.466
Folate		1.585	1.187	4.879	0.477–49.953				
Vitamin A		5.675	8.561	291.507	1.50E−05–5652141075.191				
Vitamin B12		−1.279	0.928	0.278	0.045–1.717				
Vitamin B6		−1.472	1.223	0.230	0.021–2.522				
Vitamin C		0.835	1.147	2.304	0.243–21.817				
Vitamin D		1.559	0.819	4.756	0.955–23.689				
Vitamin E		−1.323	0.745	0.266	0.067–1.148				

### 3.5 Categorical Analysis

**FIGURE 5:**
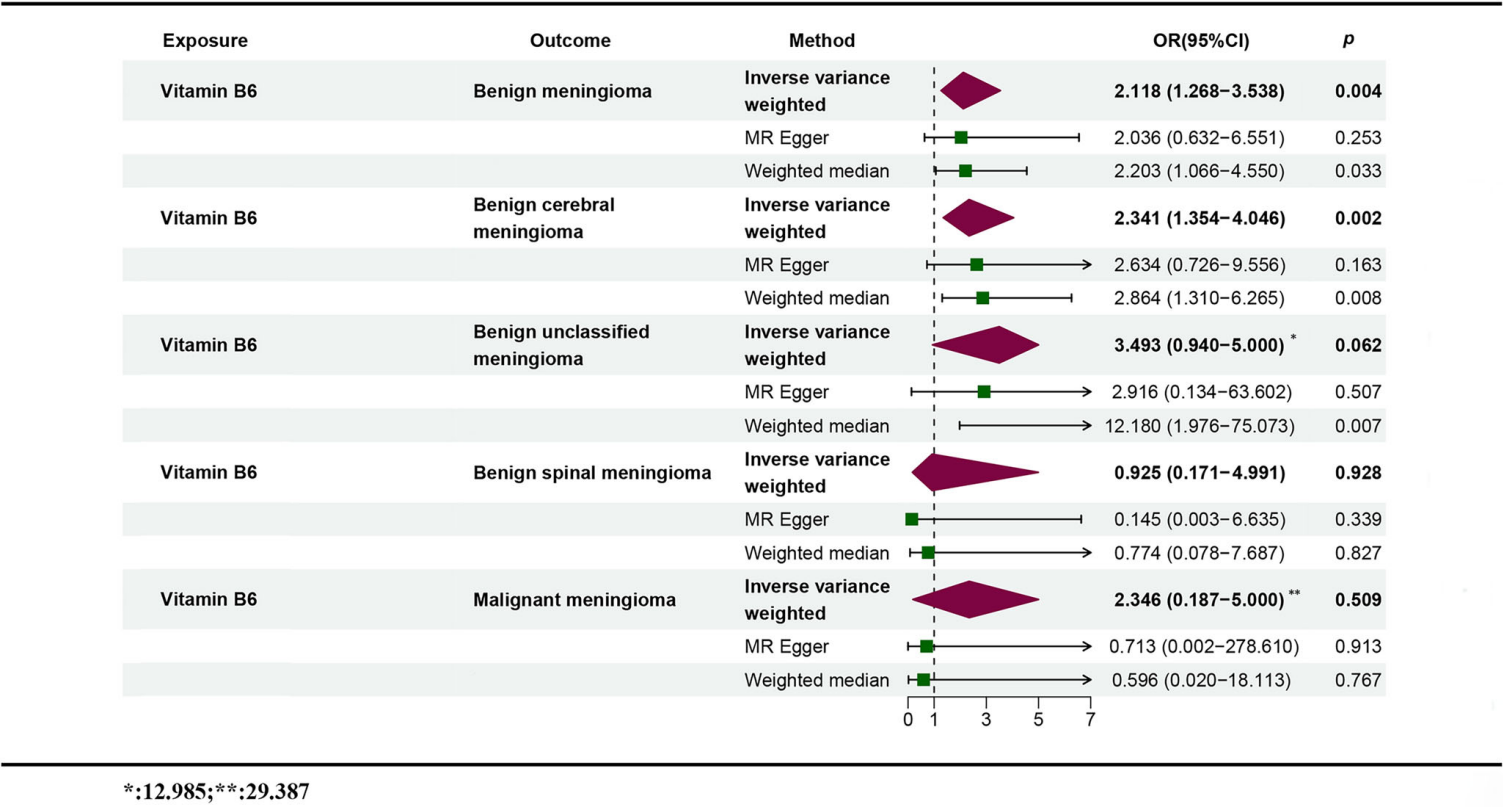
Mendelian randomization analysis of the correlation between vitamin B6 and the risk of various types of meningiomas.

**FIGURE 6:**
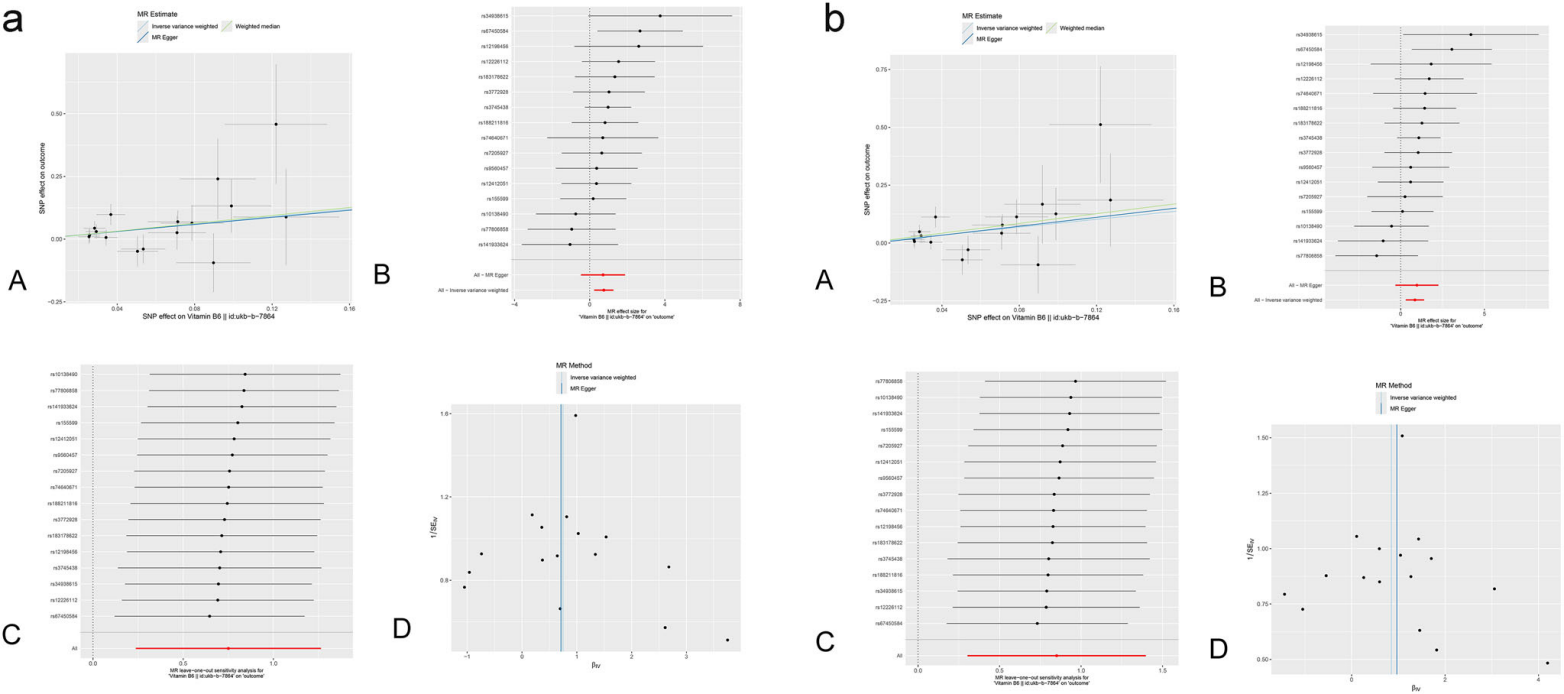
Scatter plot (A), forest plot (B), leave‐one‐out sensitivity analysis (C), and funnel plot (D) of the association of zinc with Benign meningioma (a). Scatter plot (A), Forest plot (B), Leave‐one‐out sensitivity analysis (C) and Funnel plot (D) of the association of zinc with benign cerebral meningioma (b).

To validate these findings from multiple perspectives, subsequent analyses adopted a more detailed classification standard for meningiomas, including subtypes such as benign meningiomas, benign cerebral meningiomas, benign spinal meningioma, benign unclassified meningiomas, and malignant meningiomas (Huang et al. 2025). Through two‐sample MR analysis with seven trace elements and eight nutrients as exposure factors and the aforementioned meningioma subtypes as outcome indicators, it was found that VB6 exposure increased the risk of benign meningiomas (OR = 2.118, 95% CI: 1.268–3.538, *p* = 0.004) and benign cerebral meningiomas (OR = 2.341, 95% CI: 1.354–4.046, *p* = 0.002), as shown in Figure 5. The relevant results were visually presented through scatter plots, funnel plots, forest plots, and leave‐one‐out sensitivity analysis plots (Figure 6a,b). Finally, a multivariable MR analysis was performed. The results were negative or not statistically significant.

## 4 Discussion

To the best of our knowledge, this is the first study to utilize the MR method to investigate the influence of trace elements and nutrients on meningiomas. Evidence from published GWAS data reveals a marked positive causal relationship between Zn exposure and the risk of meningiomas (OR = 1.484, 95% CI: 1.222–1.803, *p* = 6.90E‐05). In both MVMR and sensitivity analyses, the link demonstrates robustness. Notably, the link between VB6 and meningiomas is not statistically significant after Bonferroni correction, and RMR analysis also fails to reveal a noticeable causal relationship. The finding offers fresh insights into the etiology of meningiomas and highlights the intricate biological roles of trace elements and nutrients in the development of tumors.

In the MR analysis, the current study rigorously adhered to three core assumptions: SNPs are strongly associated with the exposure factor (Assumption 1), SNPs are independent of confounders (Assumption 2), and SNPs influence the outcome only through the exposure (exclusion restriction, Assumption 3). The study employed systematic sensitivity analyses for validation. Specifically, neither the MR‐Egger intercept test nor the MR‐PRESSO global test detected significant horizontal pleiotropy (p > 0.05, Table 2). These findings suggested that the selected IVs were essentially independent of potential confounders (e.g., environmental or lifestyle variables), thereby supporting Assumption 2. Concurrently, Cochran's *Q* test and LOO analysis confirmed the homogeneity and robustness of IVs. These findings precluded the possibility of SNPs directly affecting meningiomas and thus validated Assumption 3. Despite the strong support provided by these tests, it is important to acknowledge the inherent limitations of MR. The genetic instruments themselves may possess undetected pleiotropy or structural confounding. Future research should integrate multi‐omics data for multi‐layered validation to mitigate the risk of confounders and enhance the robustness of causal inferences.

In recent years, the dysregulation of trace elements and nutrient metabolism, which is a key biological feature of malignant tumors, has become a major focus in cancer research (Chen et al. 2024; H. Wang et al. 2021). Existing studies have provided a clear explanation of the molecular mechanisms through which multiple trace elements contribute to tumorigenesis and tumor progression (Haşimoğlu et al. 2022). Being an essential trace element, Zn occupies a key position in the metabolic processes of human physiology (Halsted and Prasad 1960). It is vital for biochemical and cellular functions (MacDonald 2000). Zn contributes to enzymes that participate in nucleic acid and protein synthesis and is also involved in critical activities such as the immune and nervous systems (Prakash et al. 2015). Relevant research indicates that the disruption of Zn ion homeostasis can lead to abnormal cell structure or the loss of basic physiological functions, thereby contributing to the development of various diseases, like cardiovascular diseases (Begum et al. 2022). In tumor biology, it has been proven that Zn homeostasis dysregulation and malignant tumors are interconnected via a bidirectional regulatory relationship. Tumor microenvironments may lead to Zn metabolic imbalance, and disrupted Zn distribution participates in the carcinogenic process by controlling mechanisms such as cell proliferation and apoptosis (Chen et al. 2024).

It has been suggested that Zn ions have heterogeneous effects across various cancer types, mainly due to their dual role in cancer progression, which can be divided into three main areas. First, the effect of Zn ions varies across different types of cancer cells. For example, in prostate cancer, they play an antitumor role (Costello and Franklin 2006; Fontana et al. 2023), whereas in breast cancer, pancreatic cancer, and other cancers, they promote tumor growth (M. Liu et al. 2020; Taylor et al. 2008). Second, the varying concentrations of Zn ions could be a key factor contributing to the differences in outcomes (Xue et al. 2019; R. Zhang et al. 2020). At physiological concentrations (12–20 µmol/L), the supplementation of Zn ions can meet the biosynthetic and catabolic demands of tumor cells, thereby promoting tumor growth (R. Zhang et al. 2020). However, high levels may trigger oxidative stress in tumor cells, influencing DNA damage and repair pathways, thus exerting an inhibitory effect on tumor growth (Xue et al. 2019). It has been observed that serum Zn levels decrease in patients with lung cancer, head and neck tumors, breast cancer, prostate cancer, liver cancer, and others. Moreover, its concentration in tumor tissues is also affected (Hrabeta et al. 2016). Ultimately, Zn ions might exert varying effects on the activation of immune cells and stromal cells in the tumor microenvironment. When immune‐suppressive cells are activated by them, it may result in tumor promotion (Lee et al. 2008), while the activation of tumor‐killing cell subsets could induce anti‐tumor effects (D. Yang et al. 2024).

This study, through bidirectional two‐sample MR and MVMR analyses, finds that Zn is an independent risk factor for meningiomas. This result presents an interesting contrast to the findings of previous observational studies. According to Yoshida et al. (1993), the Zn concentration in meningioma tissues is lower than in other brain tumor tissues. Haşimoğlu et al. (Haşimoğlu et al. 2022) have discovered that the serum Zn levels in meningioma patients are lower than those in the healthy control group. This apparent contradiction may actually reflect the complex biological link between Zn and meningioma. This contradiction warrants in‐depth exploration from multiple perspectives. First, traditional observational studies mostly use cross‐sectional designs. These studies can only capture Zn level status at a specific point in time and are susceptible to reverse causality and confounders. The current study applied the MR approach, using genetic variants as IVs. These variants are established at fertilization and remain largely unaffected by acquired environmental or disease states. This theoretically allows for a more effective inference of the long‐term causal association between Zn exposure and meningioma. However, the MR method also has its inherent limitations (Lee et al. 2008; Xue et al. 2019). It relies on IVs that must strictly meet the three core assumptions: independence, relevance, and exclusivity. In practice, it is difficult to completely exclude horizontal pleiotropy and weak instrumental variable bias. Therefore, while this study suggests a possible causal relationship between Zn exposure and meningioma risk at the genetic level, further verification is needed through large‐sample prospective cohorts and mechanistic experiments. Second, these differing results may reflect the dynamic changes in Zn metabolism during disease development. MR uncovers the influence of early or long‐term Zn exposure on disease risk, whereas the low Zn levels observed in clinical studies might reflect metabolic disruptions occurring during tumor progression. Specifically, high levels of Zn exposure may promote the initial formation of tumors by affecting processes such as cell proliferation, apoptosis, and DNA repair. The extensive consumption of Zn by tumor cells may lead to a reduction in Zn levels in local tissues and serum. Moreover, similar dynamic patterns have been reported in other cancer studies (Bergmann et al. 2018; J. Wang et al. 2020). Furthermore, Zn, as an essential trace element, can influence meningioma development and progression through multiple molecular pathways. According to the molecular pathway hypothesis, Zn acts as a cofactor for matrix metalloproteinases (MMPs), which promote extracellular matrix degradation and angiogenesis by activating Zn‐dependent enzymes, such as MMP‐2 and MMP‐9. This enhances the invasiveness and recurrence tendency of meningioma (Haşimoğlu et al. 2022). Conversely, Zn finger proteins (e.g., ZEB1 or the Snail family) are Zn‐dependent transcription factors that may regulate the epithelial‐mesenchymal transition (EMT) pathway (J. Wang et al. 2020). This process gives meningioma cells the ability to migrate and invade. Additionally, Zn may also shape the pro‐tumor or anti‐tumor microenvironment by affecting tumor‐associated macrophages (TAMs) or T‐cell activity (Costello and Franklin 2006; M. Liu et al. 2020). These findings provide new evidence and research insights for understanding the link of Zn with meningiomas, while also offering some guidance for dietary management and trace element supplementation in meningioma patients.

VB6 is a water‐soluble vitamin that can serve as a coenzyme, catalyzing more than 150 enzymes and regulating the metabolism and synthesis of proteins, carbohydrates, lipids, heme, and crucial bioactive metabolites (Bird 2018). It is engaged in one‐carbon metabolism, a process that can change DNA methylation levels. DNA methylation changes may be crucial in the development and progression of tumors, with abnormal DNA methylation observed in colon cancer, gastric cancer, cervical cancer, prostate cancer, and breast cancer (Mahmoud and Ali 2019). It is noteworthy that VB6, as a key coenzyme in one‐carbon metabolism, exhibits tissue‐specific anticancer effects. A substantial body of evidence indicates that a high intake of dietary VB6 is linked to a reduced risk of cancer (Choi and Friso 2012). However, as research continues to deepen, there is also evidence indicating that excessive intake of VB6 may increase the risk of developing certain tumors, like lung cancer (Brasky et al. 2017). Although an initial MR analysis indicated a positive causal association between VB6 and meningiomas, this association lost statistical significance after Bonferroni correction. This may suggest that the initial association was a false positive or that there is a weak but biologically plausible link. The results may be limited by insufficient statistical power. First, the moderate sample size of the VB6 GWAS (*n* = 64,979) and the limited number of SNPs available for analysis resulted in relatively weak explanatory power of the constructed IVs for the exposure. This reduces the ability to detect small effects. Second, the number of meningioma cases in this study was only 1605. The statistical power of MR analysis largely depends on the sample size of the outcome event. Thus, even if a true but weak causal relationship exists, the current sample size may not provide sufficient statistical power. Under the current conditions, this study does not exclude the potential association between VB6 and meningiomas. Future studies need to validate the causal relationship utilizing larger‐scale meningioma GWAS data.

Through MR analysis, this study explores the causal link of trace elements and nutrients with meningioma from a genetic standpoint, applying multiple statistical methods and sensitivity analyses to ensure the reliability of the findings, in order to offer scientifically reasonable suggestions for the clinical management and prevention of meningioma. However, this study has certain limitations. First, all of the GWAS datasets utilized are derived from European populations. This restricts the applicability of the results to other ethnic groups, such as Asian or African populations. Significant differences in the genetic backgrounds of non‐European populations (e.g., variations in SNP frequencies) may affect the validity of IVs and the estimation of effect sizes. Concurrently, differences in environmental factors, such as dietary patterns and trace element exposure levels, may act as confounders or effect modifiers, further influencing how trace elements and nutrients are linked to meningiomas. Future studies must integrate multi‐ethnic GWAS data to evaluate ethnic‐specific or universal effects. Second, since MR analysis operates under the assumption of linear link, it cannot assess nonlinear associations between exposure and outcome. Finally, the MR analysis method calculates the causal link between exposure and outcome from a theoretical perspective. While this exploratory investigation provides a certain theoretical foundation, further investigation into the pathological and physiological mechanisms of the disease is required to confirm the findings, ensuring better application to clinical practice in the future.

## 5 Conclusion

In conclusion, this study employs MR analysis to uncover and validate the causal relationship between Zn exposure and the risk of meningioma. This conclusion may provide new perspectives for subsequent mechanistic studies. Additionally, it could inspire further exploration and validation of the scientific basis and clinical strategies regarding the application of trace elements and nutrients in the prevention and treatment of meningiomas.

## Author Contributions

**Yihong Hao**: writing – original draft, writing – review and editing, conceptualization, methodology, investigation, and formal analysis. **Jieqiong Ren**: conceptualization and methodology. **Jie Han**: investigation and formal analysis. **Dajiang Hao**: funding acquisition, resources, supervision.

## Conflicts of Interest

The authors declare no conflict of interest.

## Peer Review

The peer review history for this article is available at https://publons.com/publon/10.1002/brb3.70852.

## Supporting information




**Supplementary Material**: brb370852‐sup‐0001‐SuppMatt.xlsx

**Supplementary Material**: brb370852‐sup‐0002‐SuppMatt.docx

## Data Availability

All data generated or analyzed during this study are included in this published article and its supplementary information files.

## References

[brb370852-bib-0001] Begum, F. , H. M. Me , and M. Christov . 2022. “The Role of Zinc in Cardiovascular Disease.” Cardiology in Review 30, no. 2: 100–108. 35119422 10.1097/CRD.0000000000000382

[brb370852-bib-0002] Bergmann, C. , L. M. Guay‐Woodford , P. C. Harris , S. Horie , D. J. M. Peters , and V. E. Torres . 2018. “Polycystic Kidney Disease.” Nature Reviews Disease Primers 4, no. 1: 50. 10.1038/s41572-018-0047-y PMC6592047 30523303

[brb370852-bib-0003] Bird, R. P. 2018. “The Emerging Role of Vitamin B6 in Inflammation and Carcinogenesis.” Advances in Food and Nutrition Research 83: 151–194. 29477221 10.1016/bs.afnr.2017.11.004

[brb370852-bib-0004] Bowden, J. , F. Del Greco M , C. Minelli , G. Davey Smith , N. Sheehan , and J. Thompson . 2017. “A Framework for the Investigation of Pleiotropy in Two‐Sample Summary Data Mendelian Randomization.” Statistics in Medicine 36, no. 11: 1783–1802. 28114746 10.1002/sim.7221 PMC5434863

[brb370852-bib-0005] Bowden, J. , and M. V. Holmes . 2019. “Meta‐Analysis and Mendelian Randomization: A Review.” Research Synthesis Methods 10, no. 4: 486–496. 30861319 10.1002/jrsm.1346 PMC6973275

[brb370852-bib-0006] Brasky, T. M. , E. White , and C. L. Chen . 2017. “Long‐Term, Supplemental, One‐Carbon Metabolism‐Related Vitamin B Use in Relation to Lung Cancer Risk in the Vitamins and Lifestyle (VITAL) Cohort.” Journal of Clinical Oncology 35, no. 30: 3440–3448. 28829668 10.1200/JCO.2017.72.7735 PMC5648175

[brb370852-bib-0007] Burgess, S. , J. Bowden , T. Fall , E. Ingelsson , and S. G. Thompson . 2017. “Sensitivity Analyses for Robust Causal Inference From Mendelian Randomization Analyses With Multiple Genetic Variants.” Epidemiology 28, no. 1: 30–42. 27749700 10.1097/EDE.0000000000000559 PMC5133381

[brb370852-bib-0008] Burgess, S. , N. M. Davies , and S. G Thompson . 2016. “Bias Due to Participant Overlap in Two‐Sample Mendelian Randomization.” Genetic Epidemiology 40, no. 7: 597–608. 27625185 10.1002/gepi.21998 PMC5082560

[brb370852-bib-0009] Bycroft, C. , C. Freeman , D. Petkova , et al. 2018. “The UK Biobank Resource With Deep Phenotyping and Genomic Data.” Nature 562, no. 7726: 203–209. 30305743 10.1038/s41586-018-0579-z PMC6786975

[brb370852-bib-0010] Cao, Z. , S. Zhao , T. Wu , et al. 2024. “Genetic Information Supports a Causal Relationship Between Trace Elements, Inflammatory Proteins, and COPD: Evidence From a Mendelian Randomization Analysis.” Frontiers in Nutrition 11: 1430606. 39206312 10.3389/fnut.2024.1430606 PMC11349556

[brb370852-bib-0011] Chen, B. , P. Yu , W. N. Chan , et al. 2024. “Cellular Zinc Metabolism and Zinc Signaling: From Biological Functions to Diseases and Therapeutic Targets.” Signal Transduction and Targeted Therapy 9, no. 1: 6. 38169461 10.1038/s41392-023-01679-y PMC10761908

[brb370852-bib-0012] Choi, S. W. , and S. Friso . 2012. “Vitamins B6 and Cancer.” Sub‐Cellular Biochemistry 56: 247–264. 22116703 10.1007/978-94-007-2199-9_13

[brb370852-bib-0013] Choudhury, A. , J. T. Brandenburg , T. Chikowore , et al. 2022. “Meta‐Analysis of Sub‐Saharan African Studies Provides Insights Into Genetic Architecture of Lipid Traits.” Nature Communications 13, no. 1: 2578. 10.1038/s41467-022-30098-w PMC9095599 35546142

[brb370852-bib-0014] Christine, M. , H. Marco , R. Karl , et al. 2008. “Meningioma.” Critical Reviews in Oncology/Hematology 67, no. 2: 153–171. 18342535 10.1016/j.critrevonc.2008.01.010

[brb370852-bib-0015] Costello, L. C. , and R. B Franklin . 2006. “The Clinical Relevance of the Metabolism of Prostate Cancer; Zinc and Tumor Suppression: Connecting the Dots.” Molecular Cancer 5: 17. 16700911 10.1186/1476-4598-5-17 PMC1481516

[brb370852-bib-0016] Davies, N. M. , M. V. Holmes , and G. Davey Smith . 2018. “Reading Mendelian Randomisation Studies: A Guide, Glossary, and Checklist for Clinicians.” BMJ 362: k601. 30002074 10.1136/bmj.k601 PMC6041728

[brb370852-bib-0017] Domingues, P. H. , C. Teodósio , Á. Otero , et al. 2014. “The Protein Expression Profile of Meningioma Cells Is Associated With Distinct Cytogenetic Tumour Subgroups.” Neuropathology and Applied Neurobiology 41, no. 3: 319–332. 10.1111/nan.12127 24612434

[brb370852-bib-0018] Evans, D. M. , G. Zhu , V. Dy , et al. 2013. “Genome‐Wide Association Study Identifies Loci Affecting Blood Copper, Selenium and Zinc.” Human Molecular Genetics 22, no. 19: 3998–4006. 23720494 10.1093/hmg/ddt239 PMC3766178

[brb370852-bib-0019] FinnGen: An Expedition Into Genomics and Medicine . n.d. FINNGEN. https://www.finngen.fi/en.

[brb370852-bib-0020] Flint‐Richter, P. , L. Mandelzweig , B. Oberman , and S. Sadetzki . 2011. “Possible Interaction Between Ionizing Radiation, Smoking, and Gender in the Causation of Meningioma.” Neuro‐Oncology 13, no. 3: 345–352. 21339193 10.1093/neuonc/noq201 PMC3064606

[brb370852-bib-0021] Fontana, F. , M. Anselmi , and P Limonta . 2023. “Unraveling the Peculiar Features of Mitochondrial Metabolism and Dynamics in Prostate Cancer.” Cancers 15, no. 4: 1192. 36831534 10.3390/cancers15041192 PMC9953833

[brb370852-bib-0022] Goldbrunner, R. , P. Stavrinou , M. D. Jenkinson , et al. 2021. “EANO Guideline on the Diagnosis and Management of Meningiomas.” Neuro‐Oncology 23, no. 11: 1821–1834. 34181733 10.1093/neuonc/noab150 PMC8563316

[brb370852-bib-0023] Halsted, J. A. , and A. S. Prasad . 1960. “Syndrome of Iron Deficiency Anemia, Hepatosplenomegaly, Hypogonadism, Dwarfism and Geophagia.” Transactions of the American Clinical and Climatological Association 72: 130–149. 13710935 PMC2249139

[brb370852-bib-0024] Haşimoğlu, Z. , Z. Erbayraktar , E. Özer , S. Erbayraktar , and T. Erkmen . 2022. “Quantitative Analysis of Serum Zinc Levels in Primary Brain Tumor Patients.” Biological Trace Element Research 200, no. 2: 568–573. 33826072 10.1007/s12011-021-02698-y

[brb370852-bib-0025] Hrabeta, J. , T. Eckschlager , M. Stiborova , Z. Heger , S. Krizkova , and V. Adam . 2016. “Zinc and Zinc‐Containing Biomolecules in Childhood Brain Tumors.” Journal of Molecular Medicine 94, no. 11: 1199–1215. 27638340 10.1007/s00109-016-1454-8

[brb370852-bib-0026] Huang, J. W. , Y. F. Wang , Y. Q. Hu , H. Y. He , S. Q. Gao , and Y. Guo . 2025. “Breast Cancer and Meningioma Risk: A Mendelian Randomization Study.” Brain and Behavior 15, no. 3: e70422. 40083313 10.1002/brb3.70422 PMC11907105

[brb370852-bib-0027] Jayaraman, A. K. , and S Jayaraman . 2011. “Increased Level of Exogenous Zinc Induces Cytotoxicity and Up‐Regulates the Expression of the ZnT‐1 Zinc Transporter Gene in Pancreatic Cancer Cells.” The Journal of Nutritional Biochemistry 22, no. 1: 79–88. 20392624 10.1016/j.jnutbio.2009.12.001

[brb370852-bib-0028] Kurilshikov, A. , C. Medina‐Gomez , R. Bacigalupe , et al. 2021. “Large‐Scale Association Analyses Identify Host Factors Influencing human Gut Microbiome Composition.” Nature Genetics 53, no. 2: 156–165. 33462485 10.1038/s41588-020-00763-1 PMC8515199

[brb370852-bib-0029] Kurki, M. I. , J. Karjalainen , P. Palta , et al. 2023. “FinnGen Provides Genetic Insights From a Well‐Phenotyped Isolated Population.” Nature 613, no. 7944: 508–518. 36653562 10.1038/s41586-022-05473-8 PMC9849126

[brb370852-bib-0030] Lee, W. , D. Cui , M. Czesnikiewicz‐Guzik , et al. 2008. “Age‐Dependent Signature of Metallothionein Expression in Primary CD4 T Cell Responses Is Due to Sustained Zinc Signaling.” Rejuvenation Research 11, no. 6: 1001–1011. 19072254 10.1089/rej.2008.0747 PMC2848531

[brb370852-bib-0031] Li, P. , H. Wang , L. Guo , et al. 2022. “Association Between Gut Microbiota and Preeclampsia‐Eclampsia: A Two‐Sample Mendelian Randomization Study.” BMC Medicine 20, no. 1: 443. 36380372 10.1186/s12916-022-02657-x PMC9667679

[brb370852-bib-0032] Liu, M. , Y. Zhang , J. Yang , et al. 2020. “ZIP4 Increases Expression of Transcription Factor ZEB1 to Promote Integrin α3β1 Signaling and Inhibit Expression of the Gemcitabine Transporter ENT1 in Pancreatic Cancer Cells.” Gastroenterology 158, no. 3: 679–692.e1. 31711924 10.1053/j.gastro.2019.10.038 PMC7837454

[brb370852-bib-0033] Liu, X. , X. Qi , R. Han , T. Mao , and Z. Tian . 2023. “Gut Microbiota Causally Affects Cholelithiasis: A Two‐Sample Mendelian Randomization Study.” Frontiers in Cellular and Infection Microbiology 13: 1253447. 37876873 10.3389/fcimb.2023.1253447 PMC10591199

[brb370852-bib-0034] MacDonald, R. S. 2000. “The Role of Zinc in Growth and Cell Proliferation.” Journal of Nutrition 130, no. 5S: Suppl 1500s–8s. 10.1093/jn/130.5.1500S 10801966

[brb370852-bib-0035] Mahmoud, A. , and M. M Ali . 2019. “Methyl Donor Micronutrients That Modify DNA Methylation and Cancer Outcome.” Nutrients 11, no. 3: 608. 30871166 10.3390/nu11030608 PMC6471069

[brb370852-bib-0036] Nazem, A. A. , J. Ruzevick , and M. J. Ferreira Jr . 2020. “Advances in Meningioma Genomics, Proteomics, and Epigenetics: Insights Into Biomarker Identification and Targeted Therapies.” Oncotarget 11, no. 49: 4544–4553. 33346248 10.18632/oncotarget.27841 PMC7733625

[brb370852-bib-0037] Ogasawara, C. , B. D. Philbrick , and D. C. Adamson . 2021. “Meningioma: A Review of Epidemiology, Pathology, Diagnosis, Treatment, and Future Directions.” Biomedicines 9, no. 3: 319. 33801089 10.3390/biomedicines9030319 PMC8004084

[brb370852-bib-0038] Ostrom, Q. T. , H. Gittleman , P. Liao , et al. 2017. “CBTRUS Statistical Report: Primary Brain and Other central Nervous System Tumors Diagnosed in the United States in 2010‐2014.” Neuro‐Oncology 19, no. suppl_5: v1–v88. 29117289 10.1093/neuonc/nox158 PMC5693142

[brb370852-bib-0039] Ostrom, Q. T. , M. Price , C. Neff , et al. 2022. “CBTRUS Statistical Report: Primary Brain and Other Central Nervous System Tumors Diagnosed in the United States in 2015‐2019.” Neuro‐Oncology 24, no. Suppl 5: v1–v95. 36196752 10.1093/neuonc/noac202 PMC9533228

[brb370852-bib-0040] Palmer, T. M. , D. A. Lawlor , R. M. Harbord , et al. 2012. “Using Multiple Genetic Variants as Instrumental Variables for Modifiable Risk Factors.” Statistical Methods in Medical Research 21, no. 3: 223–242. 21216802 10.1177/0962280210394459 PMC3917707

[brb370852-bib-0041] Pierce, B. L. , and S. Burgess . 2013. “Efficient Design for Mendelian Randomization Studies: Subsample and 2‐Sample Instrumental Variable Estimators.” American Journal of Epidemiology 178, no. 7: 1177–1184. 23863760 10.1093/aje/kwt084 PMC3783091

[brb370852-bib-0042] Prakash, A. , K. Bharti , and A. B. Majeed . 2015. “Zinc: Indications in Brain Disorders.” Fundamental and Clinical Pharmacology 29, no. 2: 131–149. 25659970 10.1111/fcp.12110

[brb370852-bib-0043] Sanderson, E. 2021. “Multivariable Mendelian Randomization and Mediation.” Cold Spring Harbor Perspectives in Medicine 11, no. 2: a038984. 32341063 10.1101/cshperspect.a038984 PMC7849347

[brb370852-bib-0044] Sanderson, E. , G. Davey Smith , F. Windmeijer , and J. Bowden . 2019. “An Examination of Multivariable Mendelian Randomization in the Single‐Sample and Two‐Sample Summary Data Settings.” International Journal of Epidemiology 48, no. 3: 713–727. 30535378 10.1093/ije/dyy262 PMC6734942

[brb370852-bib-0045] Sanderson, E. , G. D. Smith , F. Windmeijer , and J. Bowden . 2020. “Corrigendum to: An Examination of Multivariable Mendelian Randomization in the Single‐Sample and Two‐Sample Summary Data Settings.” International Journal of Epidemiology 49, no. 3: 1057. 32529219 10.1093/ije/dyaa101 PMC7394940

[brb370852-bib-0046] Skrivankova, V. W. , R. C. Richmond , B. A. R. Woolf , et al. 2021. “Strengthening the Reporting of Observational Studies in Epidemiology Using Mendelian Randomization: The STROBE‐MR Statement.” JAMA 326, no. 16: 1614–1621. 34698778 10.1001/jama.2021.18236

[brb370852-bib-0047] Su, T. , X. Yin , J. Ren , Y. Lang , W. Zhang , and L. Cui . 2023. “Causal Relationship Between Gut Microbiota and Myasthenia Gravis: A Bidirectional Mendelian Randomization Study.” Cell & Bioscience 13, no. 1: 204. 37936124 10.1186/s13578-023-01163-8 PMC10629094

[brb370852-bib-0048] Taylor, K. M. , P. Vichova , N. Jordan , S. Hiscox , R. Hendley , and R. I. Nicholson . 2008. “ZIP7‐Mediated Intracellular Zinc Transport Contributes to Aberrant Growth Factor Signaling in Antihormone‐Resistant Breast Cancer Cells.” Endocrinology 149, no. 10: 4912–4920. 18583420 10.1210/en.2008-0351

[brb370852-bib-0049] Wang, H. , D. Lin , Q. Yu , et al. 2021. “A Promising Future of Ferroptosis in Tumor Therapy.” Frontiers in Cell and Developmental Biology 9: 629150. 34178977 10.3389/fcell.2021.629150 PMC8219969

[brb370852-bib-0050] Wang, J. , H. Zhao , Z. Xu , and X. Cheng . 2020. “Zinc Dysregulation in Cancers and Its Potential as a Therapeutic Target.” Cancer Biology & Medicine 17, no. 3: 612–625. 32944394 10.20892/j.issn.2095-3941.2020.0106 PMC7476080

[brb370852-bib-0051] Xia, D. , J. Wang , X. Zhao , T. Shen , L. Ling , and Y. Liang . 2023. “Association Between Gut Microbiota and Benign Prostatic Hyperplasia: A Two‐Sample Mendelian Randomization Study.” Frontiers in Cellular and Infection Microbiology 13: 1248381. 37799337 10.3389/fcimb.2023.1248381 PMC10548216

[brb370852-bib-0052] Xue, Y. N. , B. B. Yu , Y. N. Liu , et al. 2019. “Zinc Promotes Prostate Cancer Cell Chemosensitivity to Paclitaxel by Inhibiting Epithelial‐Mesenchymal Transition and Inducing Apoptosis.” Prostate 79, no. 6: 647–656. 30714183 10.1002/pros.23772

[brb370852-bib-0053] Yang, D. , T. Tian , X. Li , et al. 2024. “ZNT1 and Zn 2+ Control TLR4 and PD‐L1 Endocytosis in Macrophages to Improve Chemotherapy Efficacy Against Liver Tumor.” Hepatology 80, no. 2: 312–329. 37816045 10.1097/HEP.0000000000000629

[brb370852-bib-0054] Yang, H. , Y. Ding , and L. Chen . 2007. “Effect of Trace Elements on Retinopathy of Prematurity.” Journal of Huazhong University of Science and Technology. Medical Sciences = Hua Zhong Ke Ji Da Xue Xue Bao. Yi Xue Ying De Wen Ban = Huazhong Keji Daxue Xuebao. Yixue Yingdewen Ban 27, no. 5: 590–592. 18060643 10.1007/s11596-007-0529-8

[brb370852-bib-0055] Yoshida, D. , Y. Ikeda , and S Nakazawa . 1993. “Quantitative Analysis of Copper, Zinc and Copper/Zinc Ratio in Selected Human Brain Tumors.” Journal of Neuro‐Oncology 16, no. 2: 109–115. 8289088 10.1007/BF01324697

[brb370852-bib-0056] Zhang, R. , G. Zhao , H. Shi , et al. 2020. “Zinc Regulates Primary Ovarian Tumor Growth and Metastasis Through the Epithelial to Mesenchymal Transition.” Free Radical Biology and Medicine 160: 775–783. 32927017 10.1016/j.freeradbiomed.2020.09.010 PMC7704937

[brb370852-bib-0057] Zhang, W. , J. Jiang , Y. He , et al. 2022. “Association Between Vitamins and Risk of Brain Tumors: A Systematic Review and Dose‐Response Meta‐Analysis of Observational Studies.” Frontiers in Nutrition 9: 935706. 35967781 10.3389/fnut.2022.935706 PMC9372437

[brb370852-bib-0058] Zhang, Z. , S. Wang , F. Ren et al. 2023. “Inflammatory Factors and Risk of Meningiomas: A Bidirectional Mendelian‐Randomization Study.” Frontiers in Neuroscience 17: 1186312. 37425011 10.3389/fnins.2023.1186312 PMC10325787

[brb370852-bib-0059] Zhu, R. C. , F. F. Li , Y. Q. Wu , Q. Y. Yi , and X. F. Huang . 2023. “Minimal Effect of Sleep on the Risk of Age‐Related Macular Degeneration: A Mendelian Randomization Study.” Frontiers in Aging Neuroscience 15: 1159711. 37671084 10.3389/fnagi.2023.1159711 PMC10475584

